# Prealbumin-to-Globulin Ratio Can Predict the Chemotherapy Outcomes and Prognosis of Patients with Gastric Cancer Receiving First-Line Chemotherapy

**DOI:** 10.1155/2020/6813176

**Published:** 2020-08-05

**Authors:** Zhuo Wang, Liqun Zhang, Jingyan Wang, Yuanhe Wang, Qian Dong, Haiyan Piao, Qiwei Wang, Jingdong Zhang

**Affiliations:** ^1^Medical Oncology Department of Gastrointestinal Cancer, Liaoning Cancer Hospital & Institute, Cancer Hospital of China Medical University, No. 44 Xiaoheyan Road, Dadong District, Shenyang, 110042 Liaoning Province, China; ^2^Department of Medical Oncology, Shenyang Fifth People Hospital, Tiexi District, Shenyang, 110020 Liaoning Province, China; ^3^Department of Medical Oncology, Liaohua Hospital, Hongwei District, Liaoyang, 111003 Liaoning Province, China

## Abstract

Gastric cancer (GC) is the fifth most common cancer and the third leading cause of cancer-related mortality worldwide. Inflammation and the nutritional status of patients with GC are important factors affecting the therapeutic effect and prognosis. Inflammatory and nutrition-related markers have been shown to be prognostic factors for patients with GC. However, few studies have investigated the relationship of the prealbumin-to-globulin ratio (PGR) with the prognosis of GC patients. The objective of the present study was to examine whether pretreatment PGR is related to the prognosis and chemotherapy outcomes of in-patients with advanced GC undergoing first-line chemotherapy. We retrospectively reviewed the data of 281 patients with unresectable GC from January 2013 to January 2018. The receiver operating characteristic curve analysis determined the cut-off values for the PGR. The relationship between the PGR and chemotherapy effectiveness was evaluated using the chi-square test. Kaplan-Meier's method was used to plot progression-free survival (PFS) and overall survival (OS) curves, using multivariable Cox regression analysis to identify promising predictors of mortality. The cut-off value for the PGR was 7.21. The high-PGR (≥7.21) group had a higher disease control rate than that of the low-PGR group (93.66% vs. 78.42%, *p* < 0.001). Kaplan-Meier's analysis showed significantly higher median PFS (189 vs. 125 days, *p* < 0.001) and OS (350 vs. 288 days, *p* < 0.001) in the high-PGR group. The multivariate analyses revealed that a high PGR is an independent protective factor in patients with advanced GC, both in terms of PFS (hazard ratio [HR]: 0.672; 95% confidence interval [CI]: 0.527–0.857; *p* < 0.001) and OS (HR: 0.675; 95% CI: 0.530–0.861; *p* = 0.002). In conclusion, the prechemotherapy PGR can accurately predict the chemotherapy outcome, PFS, and OS of patients with advanced GC. Therefore, medical practitioners can utilize the PGR as a novel dependable prognostic tool to weigh the prognosis of patients with GC.

## 1. Introduction

Although gastric cancer (GC) is the fifth most common cancer, it is the third leading cause of cancer-related mortality worldwide [1]. Through surgery, systemic treatments, and growing stress on multidisciplinary evaluation and treatment of patients, the 5-year net survival rate of GC is generally in the range of 25–30%. However, the median overall survival (OS) of patients with unresectable GC is only 9–11 months with combination cytotoxic chemotherapy as the global standard of care [2]. The human epidermal growth factor receptor (HER)-2 status can be used to predict the effect of targeted therapy and the prognosis. Although the median OS of patients with HER-2-positive GC is extended to 14–16 months with addition of trastuzumab in backbone chemotherapy, patients with HER-2 gene amplification or protein overexpression account for only 20% of the overall GC population [3, 4]. The Union for International Cancer Control recommends the tumor-node-metastasis (TNM) classification for GC. However, its predictive value remains deficient, particularly for patients with advanced GC, who have lost the chance for radical surgery. Therefore, there is an increasing demand for novel predictors of the chemotherapy response and survival period in patients with unresectable GC, which will aid physicians by facilitating decision-making in identifying appropriate patients for chemotherapy programs.

The therapeutic effect and prognosis of patients with cancer may be related to their inflammatory and nutritional status. Proinflammatory chemokines and cytokines participate in the occurrence, development, recurrence, and metastasis of tumors, and reduce the effectiveness of cytotoxic drugs, ultimately affecting the prognosis [5, 6]. Additionally, patients with cancer are prone to malnutrition, which reduces their tolerance of cancer treatment and the likelihood of response, and is an independent predictor of poor outcome [7]. Among the inflammation- and nutrition-related biomarkers, relevant research has proven that the albumin-to-globulin ratio (AGR), fibrinogen-to-albumin ratio (FAR), neutrophil-to-lymphocyte ratio (NLR), lymphocyte-to-monocyte ratio (LMR), and platelet-to-lymphocyte ratio (PLR) have a significant prognostic value in patients with cancer receiving cytotoxic drug therapy [8–12].

Albumin and globulin alone can develop into indicators of the patients' prognosis. However, albumin and globulin test results are affected by various factors, including physiological and pathological changes. Studies have combined albumin and globulin as predictors to minimize the impact of the inaccuracy of an isolated value on the result. Numerous studies have reported a relationship between the pretreatment AGR and the treatment response in patients with GC [13–15]. Compared with albumin, prealbumin has a shorter half-life and a lower plasma concentration [16]. Even minor changes in the nutritional status can be detected by the prealbumin level, which may make prealbumin a potential prognostic factor superior to albumin [17]. In a recent study, it was found that the prealbumin-to-globulin ratio (PGR) can indicate the chemotherapy efficacy and the prognosis of patients with cancer [18]. However, the analysis of the PGR performed in patients with GC was relatively limited.

The main objective of this study was to evaluate whether the pretreatment PGR could develop into a marker of chemotherapy response and a prognostic marker for the progression-free survival (PFS) and OS in patients with unresectable GC receiving first-line chemotherapy. We also compared the PGR with the AGR to determine which is a better prognostic indicator. Thus, this study investigated two potential prognostic factors, the PGR and AGR.

## 2. Materials and Methods

### 2.1. Patient Selection and Follow-Up

We retrospectively reviewed the records of patients with nonresectable GC evaluated from January 2013 to January 2018 at the Cancer Hospital of the China Medical University in Shenyang City, Liaoning Province, China.

The following were the enrollment criteria: (1) chemotherapy-naive, stage III-IV unresectable gastric adenocarcinoma diagnosed by biopsy and imaging; (2) an Eastern Cooperative Oncology Group (ECOG) performance status score of 0–2; (3) no severe complications that may hinder patients from receiving chemotherapy; (4) completed at least two cycles of chemotherapy; and (5) completed all required blood tests within 1 week before the initial first-line chemotherapy. We excluded patients with kidney or liver diseases other than tumors and those with severe chronic or acute inflammatory diseases.

Radiographic assessments were performed at baseline and every 6–8 weeks until disease progression. After the failure of first-line chemotherapy, the patients were followed up every 8–12 weeks until they died. Variations of approximately 1 week were regarded as a permissible error.

All enrolled patients volunteered to participate in the study, which was approved by the Ethics Committee of the Cancer Hospital of China Medical University.

### 2.2. Clinical Data Collection

We collected the following data from the hospital records: the patients' characteristics before first-line chemotherapy, the location of the tumor in the stomach as determined by gastroscopy, and computed tomography (CT)-, magnetic resonance imaging (MRI)-, and positron emission tomography (PET)/CT-determined metastatic lymph nodes and organs. Peripheral blood analysis and albumin, prealbumin, globulin, hemoglobin, carcinoembryonic antigen (CEA), carbohydrate antigen 72-4 (CA72-4), and carbohydrate antigen 19-9 (CA19-9) levels were obtained within 1 week of treatment initiation. The reference ranges for plasma albumin, prealbumin, globulin, and hemoglobin were 35–50 g/L, 160–450 mg/L, 20–35 g/L, and 115–155 g/L, respectively. The accepted normal ranges of CEA, CA72-4, and CA19-9 were 0–5 ng/ml, 0–6 U/ml, and 0–37 U/ml, respectively. The PGR and AGR were defined as prealbumin (mg/L)/globulin (g/L) ratio and albumin (g/L)/globulin (g/L) ratio, respectively.

All patients received different systemic first-line chemotherapy regimens depending on their ECOG score. Patients were evaluated for therapy response after every two cycles of chemotherapy. We adopted the Response Evaluation Criteria in Solid Tumors (RECIST 1.1) to assess the patients' chemotherapy response, as follows: complete remission (CR), partial response (PR), stable disease (SD), and disease progression. The objective response rate (ORR) was calculated as CR + PR. The disease control rate (DCR) was calculated as CR + PR + SD. The PFS was defined as the period from first-line chemotherapy until disease progression or death as a result of any cause, whichever occurred first, and the OS was defined as the period from the date of first-line chemotherapy to the date of death from any cause.

### 2.3. Data Analysis Methods

The Statistical Package for Social Sciences version 25 software (SPSS Inc., Chicago, IL, USA) was used for data analysis. We used the cut-off values of the PGR and AGR determined by a receiver operating characteristic curve analysis to divide all patients with unresectable GC into high-value and low-value groups for each factor. Fisher's exact test and the chi-square test were used to evaluate the relationship between the PGR or AGR and the patients' characteristics and therapeutic response. Kaplan-Meier's method was used to plot the PFS and OS curves, and the PFS and OS were analyzed using the log-rank test. Pearson's correlation analysis was employed to assess the linear correlation between the prognostic factors and the PFS and OS. Univariate and multivariate analyses were performed using a Cox proportional hazards model to identify potential predictors of mortality. We verified that the proportional hazards assumption was met [19]. In the proportional hazards assumption, we classified patients into two groups based on the following criteria: (1) median age of 60 years; (2) body mass index less than 18.5 or greater than 25 kg/m^2^, CEA >5 ng/mL, CA72-4 >6 U/mL, CA19-9 >37 U/mL, and hemoglobin <115 g/L; abnormal values that may be associated with a poor patient prognosis; and (3) compared with those with ECOG scores 0–1, papillary or tubular adenocarcinoma, TNM stage III, number of tumor organ metastases 0–1, and no peritoneal metastasis, patients with ECOG ≥2, poorly cohesive or mucinous adenocarcinoma, TNM stage IV, number of tumor organ metastases ≥2, and peritoneal metastasis may have a worse prognosis. Those variables that were clinically and statistically significant in the univariate analysis were included in multivariate Cox regression models. A *p* value <0.05 was considered statistically significant.

## 3. Results

### 3.1. Identification of the Optimal Cut-off Value for the PGR and AGR

**Figure 1:**
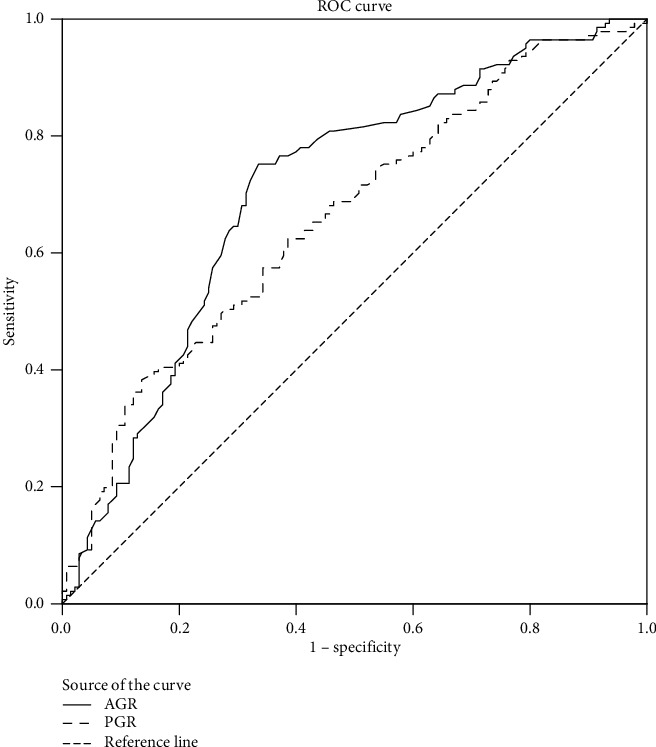
Receiver operating characteristic curve analysis of the prealbumin-to-globulin ratio (PGR) and the albumin-to-globulin ratio (AGR) in patients with unresectable gastric cancer.

The median PFS of 151 days was the state variable, the PGR and AGR were used as the test variables, and then, the cut-off values of the above two prognostic components were determined. We found that a PGR of 7.205 and an AGR of 1.455 were the strongest prognostic factors for the PFS. The area under the curve (AUC) values for the PGR and AGR were 0.660 (95% confidence interval [CI]: 0.597–0.723, sensitivity = 0.624, specificity = 0.614, *p* < 0.001) and 0.707 (95% CI: 0.646–0.769, sensitivity = 0.752, specificity = 0.664, *p* < 0.001), respectively. From the results, the AUC value of the AGR was slightly larger than that of the PGR; thus, the AGR may be better than the PGR for predicting the prognosis of patients. In this study, we set the cut-off values for the PGR and AGR to 7.21 and 1.46, respectively. Patients with a PGR ≥7.21 and an AGR ≥1.46 were categorized in the high-PGR and high-AGR groups, respectively; otherwise, they were categorized in the low-PGR and low-AGR groups, respectively (Figure 1).

### 3.2. Clinicopathological Features

A total of 281 patients with advanced GC receiving first-line chemotherapy were included in the analysis. The majority of patients were men (66.55%), and the median age of the patients was 60 years. Among all, 74.38% and 82.92% of patients had a normal body mass index and an ECOG score less than 2 points, respectively. Patients with poorly cohesive or mucinous adenocarcinoma (74.02%) and stage IV disease (80.43%) represented the highest proportions among all patients. The number of patients with more than one organ metastases was the same as that with peritoneal metastases, accounting for 32.38% of the total. SOX (oxaliplatin + S1)/CapeOX (oxaliplatin + capecitabine), FOLFOX (oxaliplatin + leucovorin +5-fluorouracil), and DCF (docetaxel + cisplatin +5-fluorouracil)/DOF (docetaxel + oxaliplatin +5-fluorouracil) were the primary chemotherapy regimens for patients receiving first-line chemotherapy, accounting for 50.53%, 17.44%, and 10.68% of the total, respectively. Based on the RECIST1.1 criteria, the ORR and DCR were 13.17% and 86.12%, respectively.  Table 1 details the characteristics of the patients.

There were some differences in terms of the histopathological and clinical parameters between the low and high groups. The analysis indicated that a low PGR (*p* = 0.006) and a low AGR (*p* = 0.001) were significantly related to a hemoglobin level <115 g/L. The probability of more than one organ metastases and peritoneal metastases was statistically higher in the low-AGR group (*p* = 0.029; Table 2).

### 3.3. Relationship between the PGR and AGR and the First-Line Chemotherapy Response

We evaluated the relationship between the PGR and AGR levels before receiving first-line chemotherapy and the treatment response. Patients in the high-PGR (93.66% vs. 78.42%, *p* < 0.001) and high-AGR (94.12% vs. 76.56%, *p* < 0.001) groups had a higher DCR than that of those in the low-groups. The high-AGR group had an ORR nearly two times higher than that of the low-AGR group (16.99% vs. 8.59%, *p* = 0.038). Although patients in the high-PGR group tended to have a higher ORR, the difference did not reach statistical significance (15.49% vs. 10.79%, *p* = 0.244). These findings indicate that the AGR may be superior to the PGR in predicting chemotherapy response (Table 3).

### 3.4. Survival Analysis according to the Pretreatment PGR and AGR

**Figure 2:**
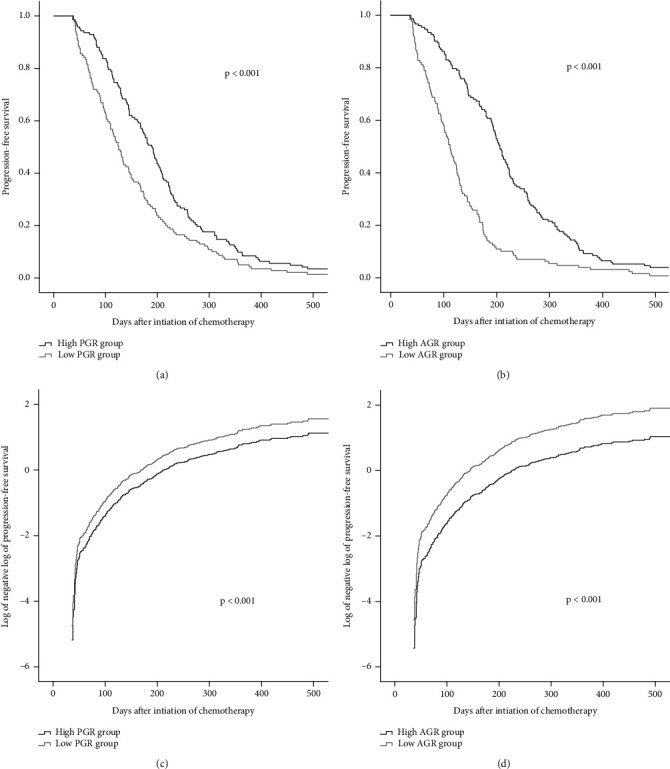
Kaplan-Meier's curves for progression-free survival of the PGR (a) and AGR (b) groups. Log of the negative log of the survival curve for progression-free survival of the PGR (c) and AGR (d) groups. PGR: prealbumin-to-globulin ratio; AGR: albumin-to-globulin ratio.

**Figure 3:**
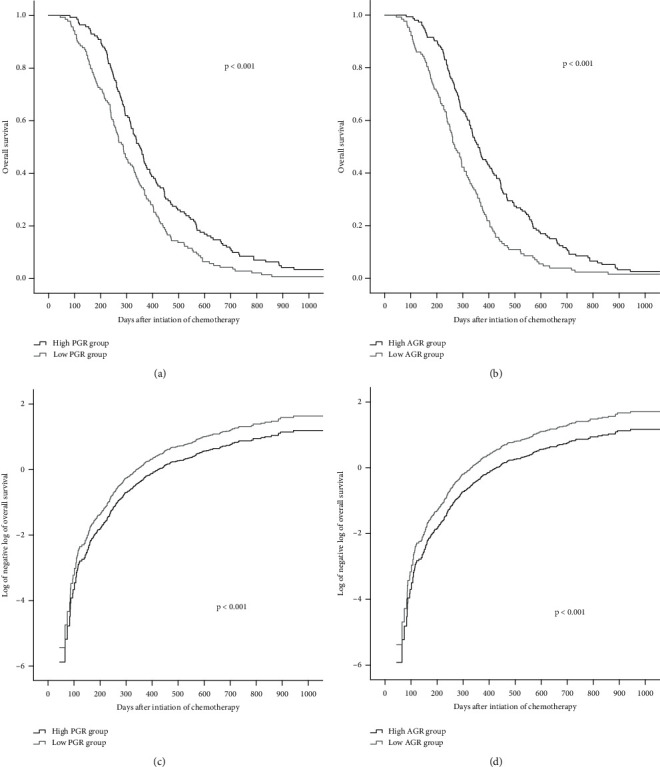
Kaplan-Meier's curve for overall survival of the PGR (a) and AGR (b) groups. Log of the negative log of the survival curve for overall survival of the PGR (c) and AGR (d) groups. PGR: prealbumin-to-globulin ratio; AGR: albumin-to-globulin ratio.

Figures 2 and 3 show the Kaplan-Meier curves for survival. The validity of the proportional hazards assumption for the model was assessed via log(−log[survival]) plots. In the whole group, the patients' median PFS and OS were 151 and 322 days, respectively. The high-PGR group had longer median PFS (189 vs. 125 days, *p* < 0.001) and median OS (350 vs. 288 days, *p* < 0.001) than those of the low-PGR group. Compared with the low-AGR group, the high-AGR group had extended median PFS (206 vs. 112 days, *p* < 0.001) and median OS (359 vs. 269 days, *p* < 0.001).

**Figure 4:**
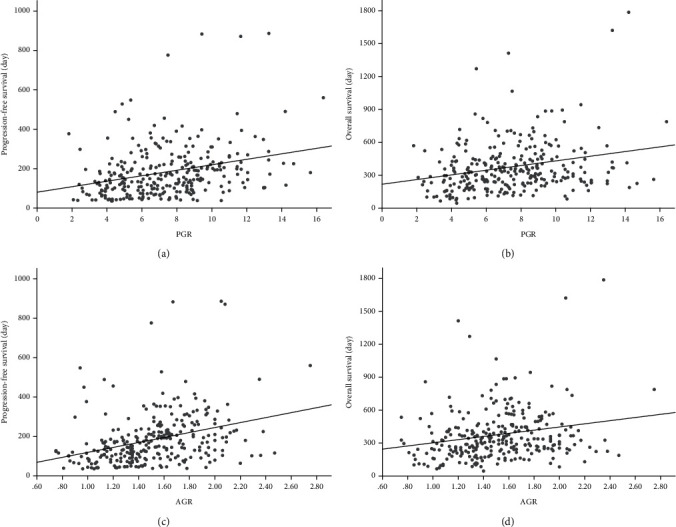
Pearson's correlation s analysis for the linear relationship between the PGR group and progression-free survival (a) and overall survival (b); Pearson's correlation s analysis for the linear relationship between the AGR group and progression-free survival (c) and overall survival (d). PGR: Prealbumin-to-globulin ratio; AGR: albumin-to-globulin ratio.

Pearson's correlation analysis indicated that the PGR and AGR values were positively correlated with the PFS and OS in patients with GC (all *p* < 0.001; Figure 4).

### 3.5. Prognostic Factors Influencing the Long-Term Survival

The univariate and multivariate analyses estimated the factors affecting the PFS and OS (Tables 4 and 5). The univariate Cox regression analyses revealed that the high-PGR group had longer PFS (hazard ratio [HR]: 0.644; 95% CI: 0.508–0.817; *p* < 0.001) and OS (HR: 0.644; 95% CI: 0.507–0.817; *p* < 0.001) compared with the low-PGR group. Analogous results were found in the high-AGR group. After adjusting for the effects of the prognostic factors in the multivariate analysis, the high-PGR group was still strongly associated with prolonged PFS (HR: 0.672; 95% CI: 0.527–0.857; *p* = 0.001) and OS (HR: 0.675; 95% CI: 0.530–0.861; *p* = 0.002), as was the high-AGR group. Regarding the other prognostic elements in the multivariate analysis, poorly cohesive or mucinous adenocarcinoma, more than one organ affected by metastasis, and CA72-4 >6 U/mL were associated with limited PFS, and poorly cohesive or mucinous adenocarcinoma, peritoneal dissemination, CEA >5 ng/mL were associated with unfavorable OS (all *p* < 0.05).

## 4. Discussion

Systemic inflammation and malnutrition are prevailing in patients with cancer. These factors have a significant impact on the patients' quality of life, treatment outcomes, diagnosis, and durability, and may increase the risk of longer duration of hospitalization, infection, treatment toxicity, and treatment costs [5, 7, 20, 21]. These effects are also manifested in patients with GC, particularly those with advanced cancer. Patients with gastrointestinal tumors are more likely to suffer from gastrointestinal obstruction and malabsorption and other complications. Hence, early detection of inflammation and malnutrition is crucial. However, medical workers tend to belittle this fact during anticancer treatment, making it imperative to appraise the inflammatory and nutritional status of patients with cancer.

Multiple studies have investigated a series of inflammatory and nutritional markers available to doctors, including the PGR, AGR, FAR, PLR, NLR, and LMR, for the prognosis of patients with different types of cancer [18, 22–26]. Among them, the AGR, FAR, PLR, NLR, and LMR were frequently applied to anticipate complications and the prognosis of patients with GC [27–31]. However, there is scant evidence on the relationship between the PGR and the prognosis of patients with GC.

In this study, we explored the prognostic and predictive role of the PGR in patients with GC receiving first-line chemotherapy. First, we found that a low pretreatment PGR was strongly associated with a hemoglobin level of <115 g/L. A previous study has shown that patients with GC who have anemia have a poor prognosis [32], which indirectly reveals that the PGR is a reliable prognostic biomarker. Next, we assessed the ability of the PGR level to predict the chemotherapy response. Patients in the high-PGR group had a higher DCR than that of those in the low-PGR groups. The high PGR value contributed to a superior ORR, but it did not achieve statistical significance. This was probably due to the small sample size. Furthermore, we found that a high PGR was significantly associated with prolonged OS and PFS in the survival analysis, and the PGR value was positively linearly correlated with the PFS and OS. Finally, the multivariate analysis revealed that the PGR was an independent prognostic indicator for the survival of patients with GC receiving chemotherapy, which further establishes a sturdy bedrock for the PGR to mature into an applicable prognostic biomarker for patients with GC. The inherent mechanisms of the PGR as a prognostic marker for patients with GC are as follows.

First, inflammatory cytokines released by inflammatory cells can cultivate an inflammatory microenvironment, which provides a hotbed for tumor growth. Therefore, chronic inflammation is a contributor to tumor cell growth, angiogenesis, development, progression, recurrence, and metastasis [5]. The level of globulin contained in the PGR may reflect the level of inflammation in patients [33]. As a chronic inflammation biochemical marker, the concentration of serum globulin gradually increases when stimulated by proinflammatory cytokines, such as tumor necrosis factor-*α*, interleukin-6, and interleukin-1b [34]. Previous studies have shown that globulin levels are associated with shorter survival in patients [35, 36].

Second, malnutrition relates intimately to a decline in the quality of life, reducing the adherence to treatment and response to therapy. As malnutrition is one of the leading causes for immunodeficiency, the nutritional status can be used to comparatively quickly assess the host's immune status [37, 38]. Protein-related malnutrition is universal in patients with advanced tumors receiving treatment and eventually leads to the collapse of the cellular and humoral immunity, which is known to play a central role in the defenses against malignant tumor cells. Malnutrition can affect the synthesis of prealbumin, one of the PGR components; thus, prealbumin can be used as a surrogate marker to mirror the nutritional status of patients [39]. Compared with albumin, prealbumin has a shorter half-life of 2 days and a smaller concentration in the body, which makes it susceptible to changes in the serum and an ideal candidate sensitive marker for predicting the prognosis of patients [40]. Earlier studies have discussed the independent prognostic role of serum prealbumin [41, 42].

Finally, globulin and prealbumin are two potentially valued elements related to the prognosis of patients with cancer. However, these parameters alone are not sufficient to predict the patients' prognosis. The globulin level is an inversely related prognostic index, while the prealbumin level is a positively related prognostic index. Integrating indicators into the PGR has improved accuracy. By calculating the ratio of globulin to prealbumin, lower values are accompanied by inflammation or/and malnutrition, which may be related to a poor prognosis. On the contrary, a higher value indicates no inflammation or/and great nutritional status, which can be associated with a favorable prognosis. Therefore, compared with globulin and prealbumin alone, the PGR probably has a better ability to distinguish patients with favorable prognosis from those with unfavorable prognosis.

This study has several strengths. We demonstrated for the first time that the PGR is a significant indicator that can independently predict the PFS and OS in patients with GC receiving first-line chemotherapy. This study was the first to use the PGR as a forecasting element of chemotherapeutic response for patients with GC.

There are also limitations to this study. First, the PGR was found not to be superior to the AGR as a prognostic indicator in this study. This might be because patients with advanced GC have gone through protracted disease history, prolonging chronic malnutrition and inflammation in these patients. Therefore, we speculate that the PGR with a shorter half-life of 2–3 days is superior to the AGR with a half-life of 21 days as a prognostic marker in early-stage patients, but further research is needed to validate this hypothesis. Second, the present was a single-center retrospective study. Hence, the usefulness of the PGR still needs to be verified by multicenter large-scale prospective studies.

## 5. Conclusions

The prechemotherapy PGR level can predict the chemotherapy effect in patients with advanced GC and is an independent predictor of the PFS and OS. It may be a reliable prognostic tool for medical practitioners.

## Figures and Tables

**Table 1:** Baseline characteristics of the patients.

Characteristics	Cases (*n* = 281)	Percentage
Sex		
Male	187	66.55%
Female	94	33.45%
Age (yr)		
≤60	159	56.58%
>60	122	43.42%
Body mass index (kg/m^2^)		
<18.5 or >25	72	25.62%
18.5–25	209	74.38%
ECOG		
0–1	233	82.92%
≥2	48	17.08%
Histological type		
Papillary, tubular,	73	25.98%
Poorly cohesive, mucinous	208	74.02%
TNM stage		
III	55	19.57%
IV	226	80.43%
Number of organs affected by metastasis		
0–1	190	67.62%
≥2	91	32.38%
Peritoneal metastasis		
Yes	91	32.38%
No	190	67.62%
First-line chemotherapy regimen		
SOX/CapeOX	142	50.53%
FOLFOX	49	17.44%
DCF/DOF	30	10.68%
Capecitabine/S-1	22	7.83%
Others	38	13.52%
Best response		
Complete remission	0	—
Partial response	37	13.17%
Stable disease	205	72.95%
Progression of disease	39	13.88%
Objective response rate	37	13.17%
Disease control rate	242	86.12%
Tumor biomarkers		
CEA >5 ng/mL	121	43.06%
CA72-4 >6 U/mL	158	56.23%
CA19-9 >37 U/mL	106	37.72%
Hemoglobin (g/L)		
<115	105	37.37%
≥115	176	62.63%
Albumin (g/L)		
<35	52	18.51%
≥35	229	81.49%
Prealbumin (mg/L)		
<160	83	29.54%
≥160	198	70.46%
Globulin (g/L)		
≤35	259	92.17%
>35	22	7.83%
PGR		
<7.21	139	49.47%
≥7.21	142	50.53%
AGR		
<1.46	128	45.55%
≥1.46	153	54.45%

ECOG: Eastern Cooperative Oncology Group; TNM: tumor-node-metastasis; SOX: oxaliplatin + S1; CapeOX: oxaliplatin + capecitabine; FOLFOX: oxaliplatin + leucovorin + 5-fluorouracil; DCF: docetaxel + cisplatin + 5-fluorouracil; DOF: docetaxel + oxaliplatin + 5-fluorouracil; CEA: carcinoembryonic antigen; CA72-4: carbonhydrate antigen 72-4; CA 19-9: carbonhydrate antigen 19-9; PGR: prealbumin-to-globulin ratio; AGR: albumin-to-globulin ratio.

**Table 2:** Relationship between the pretreatment PGR and AGR and clinicopathological factors.

	PGR			AGR		
	Low	High	*p* value	Low	High	*p* value
Sex						
Male	95	92	0.528	86	101	0.835
Female	44	50		42	52	
Age (yr)						
>60	63	59	0.523	61	61	0.190
≤60	76	83		67	92	
Body mass index (kg/m^2^)						
<18.5 or >25	40	32	0.231	30	42	0.443
18.5–25	99	110		98	111	
ECOG						
0–1	113	120	0.474	104	129	0.497
≥2	26	22		24	24	
Histological type						
Papillary, tubular	35	38	0.763	27	46	0.088
Poorly cohesive, mucinous	104	104		101	107	
TNM stage						
III	26	29	0.717	21	34	0.221
IV	113	113		107	119	
Number of organs affected by metastasis						
0–1	90	100	0.309	78	112	0.029
≥2	49	42		50	41	
Peritoneal metastasis						
Yes	52	39	0.075	50	41	0.029
No	87	103		78	112	
CEA (ng/mL)						
>5	67	54	0.085	56	65	0.831
≤5	72	88		72	88	
CA72-4 (U/mL)						
>6	79	79	0.839	76	82	0.331
≤6	60	63		52	71	
CA19-9 (U/mL)						
>37	59	47	0.106	52	54	0.359
≤37	80	95		76	99	
Hemoglobin (g/L)						
<115	63	42	0.006	61	44	0.001
≥115	76	100		67	109	

PGR: prealbumin-to-globulin ratio; AGR: albumin-to-globulin ratio; ECOG: Eastern Cooperative Oncology Group; TNM: tumor-node-metastasis; CEA: carcinoembryonic antigen; CA72-4: carbonhydrate antigen 72-4; CA 19-9: carbohydrate antigen 19-9.

**Table 3:** Treatment response to first-line chemotherapy according to the pretreatment PGR and AGR.

Response	PGR			AGR		
	High (*n* = 142)	Low (*n* = 139)	*p* value	High (*n* = 153)	Low (*n* = 128)	*p* value
Complete response	0	0		0	0	
Partial response	22	15		26	11	
Stable disease	111	94		118	87	
Progressive disease	9	30		9	30	
Objective response rate	15.49%	10.79%	0.244	16.99%	8.59%	0.038
Disease control rate	93.66%	78.42%	<0.001	94.12%	76.56%	<0.001

PGR: prealbumin to globulin ratio; AGR: albumin to globulin ratio.

**Table 4:** Correlations between survival and the PGR and other clinicopathological factors.

	Progression-free survival						Overall survival					
	Univariate analysis			Multivariate analysis			Univariate analysis			Multivariate analysis		
	Hazard ratio	95% CI	*p* value	Hazard ratio	95% CI	*p* value	Hazard ratio	95% CI	*p* value	Hazard ratio	95% CI	*p* value
Sex (male)	1.073	0.835-1.379	0.582				1.145	0.891-1.471	0.290			
Age (>60 years)	0.991	0.781-1.256	0.939				0.797	0.627-1.012	0.062	0.874	0.674-1.133	0.309
Body mass index (<18.5 or >25 kg/m^2^)	0.923	0.705-1.207	0.558				1.084	0.828-1.420	0.557			
ECOG (≥2)	1.124	0.823-1.535	0.463				1.174	0.859-1.604	0.313			
Histological type (poorly cohesive, mucinous)	1.492	1.136-1.959	0.004	1.495	1.137-1.965	0.004	1.537	1.172-2.015	0.002	1.407	1.067-1.855	0.015
TNM stage (IV)	1.206	0.897-1.622	0.214				1.368	1.017-1.839	0.038	1.186	0.855-1.643	0.306
Number of organs affected by metastasis (≥2)	1.390	1.081-1.786	0.010	1.400	1.077-1.818	0.012	1.364	1.058-1.758	0.017	1.294	0.973-1.721	0.077
Peritoneal metastasis (yes)	1.526	1.185-1.966	<0.001	1.297	0.991-1.696	0.058	1.710	1.325-2.205	<0.001	1.426	1.062-1.915	0.018
CEA (>5 ng/mL)	1.222	0.959-1.557	0.105	1.138	0.878-1.474	0.328	1.278	1.004-1.626	0.046	1.299	1.004-1.680	0.046
CA72-4 (>6 U/mL)	1.425	1.118-1.816	0.004	1.411	1.091-1.824	0.009	1.254	0.989-1.590	0.061	1.164	0.903-1.501	0.240
CA19-9 (>37 U/mL)	1.051	0.825-1.339	0.686				1.046	0.821-1.332	0.717			
Hemoglobin (<115 g/L)	0.905	0.708-1.157	0.424				0.890	0.697-1.136	0.349			
PGR ≥7.21	0.644	0.508-0.817	<0.001	0.672	0.527-0.857	0.001	0.644	0.507-0.817	<0.001	0.675	0.530-0.861	0.002

ECOG: Eastern Cooperative Oncology Group; TNM: tumor-node-metastasis; CEA: carcinoembryonic antigen; CA72-4: carbonhydrate antigen 72-4; CA 19-9: carbohydrate antigen 19-9; PGR: prealbumin-to-globulin ratio.

**Table 5:** Correlations between survival and AGR and other clinicopathological factors.

	Progression-free survival						Overall survival					
	Univariate analysis			Multivariate analysis			Univariate analysis			Multivariate analysis		
	Hazard ratio	95% CI	*p* value	Hazard ratio	95% CI	*p* value	Hazard ratio	95% CI	*p* value	Hazard ratio	95% CI	*p* value
Sex (male)	1.073	0.835-1.379	0.582				1.145	0.891-1.471	0.290			
Age (>60 years)	0.991	0.781-1.256	0.939				0.797	0.627-1.012	0.062	0.877	0.678-1.135	0.320
Body mass index (<18.5 or >25 kg/m^2^)	0.923	0.705-1.207	0.558				1.084	0.828-1.420	0.557			
ECOG (≥2)	1.124	0.823-1.535	0.463				1.174	0.859-1.604	0.313			
Histological type (poorly cohesive, mucinous)	1.492	1.136-1.959	0.004	1.355	1.028-1.787	0.031	1.537	1.172-2.015	0.002	1.352	1.023-1.787	0.034
TNM stage (IV)	1.206	0.897-1.622	0.214				1.368	1.017-1.839	0.038	1.182	0.852-1.640	0.318
Number of organs affected by metastasis (≥2)	1.390	1.081-1.786	0.010	1.341	1.030-1.746	0.029	1.364	1.058-1.758	0.017	1.299	0.977-1.728	0.072
Peritoneal metastasis (yes)	1.526	1.185-1.966	<0.001	1.239	0.947-1.621	0.118	1.710	1.325-2.205	<0.001	1.419	1.056-1.907	0.020
CEA (>5 ng/mL)	1.222	0.959-1.557	0.105	1.146	0.883-1.487	0.305	1.278	1.004-1.626	0.046	1.381	1.066-1.787	0.014
CA72-4 (>6 U/mL)	1.425	1.118-1.816	0.004	1.418	1.093-1.840	0.009	1.254	0.989-1.590	0.061	1.150	0.891-1.485	0.284
CA19-9 (>37 U/mL)	1.051	0.825-1.339	0.686				1.046	0.821-1.332	0.717			
Hemoglobin (<115 g/L)	0.905	0.708-1.157	0.424				0.890	0.697-1.136	0.349			
AGR ≥1.46	0.419	0.328-0.535	<0.001	0.454	0.353-0.583	<0.001	0.584	0.460-0.742	<0.001	0.602	0.472-0.769	<0.001

ECOG: Eastern Cooperative Oncology Group; TNM: tumor-node-metastasis; CEA: carcinoembryonic antigen; CA72-4: carbonhydrate antigen 72-4; CA 19-9: carbohydrate antigen 19-9; AGR: albumin-to-globulin ratio.

## Data Availability

We declared that materials described in the manuscript, including all relevant raw data, will be freely available to any scientist wishing to use them for noncommercial purposes, without breaching participant confidentiality.
